# Role of TGFβ-producing regulatory T cells in scleroderma and end-stage organ failure

**DOI:** 10.1016/j.heliyon.2024.e35590

**Published:** 2024-08-02

**Authors:** Kuo-Cheng Lu, Kuo-Wang Tsai, Wan-Chung Hu

**Affiliations:** a Division of Nephrology, Department of Medicine, Taipei Tzu Chi Hospital, Buddhist Tzu Chi Medical Foundation, New Taipei City, Taiwan; b Division of Nephrology, Department of Medicine, Fu Jen Catholic University Hospital, School of Medicine, Fu Jen Catholic University, New Taipei City, Taiwan; c Department of Medical Research, Taipei Tzu Chi Hospital, Buddhist Tzu Chi Medical Foundation, New Taipei City, 231, Taiwan; d Department of Clinical Pathology, Taipei Tzu Chi Hospital, Buddhist Tzu Chi Medical Foundation, New Taipei City, 231, Taiwan; e Department of Biotechnology, Ming Chuan University, Taoyuan City, 333, Taiwan

**Keywords:** Treg, TGFβ, Scleroderma, Heart failure, Liver cirrhosis, Uremia, Pulmonary fibrosis

## Abstract

Regulatory T cells (Tregs) are crucial immune cells that initiate a tolerable immune response. Transforming growth factor-beta (TGFβ) is a key cytokine produced by Tregs and plays a significant role in stimulating tissue fibrosis. Systemic sclerosis, an autoimmune disease characterized by organ fibrosis, is associated with an overrepresentation of regulatory T cells. This review aims to identify Treg-dominant tolerable host immune reactions and discuss their association with scleroderma and end-stage organ failure. End-stage organ failures, including heart failure, liver cirrhosis, uremia, and pulmonary fibrosis, are frequently linked to tissue fibrosis. This suggests that TGFβ-producing Tregs are involved in the pathogenesis of these conditions. However, the exact significance of TGFβ and the mechanisms through which it induces tolerable immune reactions during end-stage organ failure remain unclear. A deeper understanding of these mechanisms could lead to improved preventive and therapeutic strategies for these severe diseases.

## Highlights

• Tregs are essential for inducing tolerable immune responses and tissue fibrosis.
• TGFβ-producing Tregs are linked to systemic sclerosis and organ fibrosis.
• Review explores Treg-dominant immune reactions in scleroderma and organ failure.
• Understanding TGFβ mechanisms can improve treatments for organ failures.
• Tregs and TGFβ are key in the pathogenesis of heart, liver, and lung fibrosis.

## 1 Introduction

Immune reactions are categorized as immunoglobulin G-associated eradicable immunities and immunoglobulin A (IgA)-associated tolerable immunities [[1], [2], [3], [4], [5]]. CD4^+^CD25^+^ T lymphocytes (Tregs) mediate these tolerant immune responses by producing transforming growth factor-beta (TGFβ), which facilitates various tolerable immune reactions such as TH17, TH9, TH1-like, and TH3 responses. The TH17 response is triggered by TGFβ and interleukin-6, while the TH9 response is initiated by TGFβ and interleukin-4. However, Tregs do not induce immunosuppression. Instead, corticosteroids are responsible for physiological immunosuppression. TGFβ, in contrast, initiates tolerable IgA-dominant immune responses through milder regulatory mechanisms in response to chronic or severe pathogenic infections. IgA is a weak antibody that prevents severe immune reactions that could damage cells, tissues, or organs during extensive infections. Additionally, Tregs mediate tissue repair, with TGFβ serving as the master regulator of tissue fibrosis. TGFβ promotes the use of collagens and other extracellular matrices to repair wounds, tissues, or cells, thus linking tissue fibrosis to Treg-dominant tolerable immune reactions. Tissue fibrosis is a hallmark of both scleroderma and end-stage organ failure. In this review, we identify Treg-dominant tolerable host immune reactions and discuss their association with scleroderma and end-stage organ failure.

## 2 Intricate tapestry of host immunological pathways

The human immune system is a complex and intricate network of interconnected pathways designed to protect the body against various potential threats. At the heart of this immunological labyrinth lies a fundamental dichotomy: eradicable and tolerable immune responses, regulated by IgG and IgA antibody isotypes, respectively [1,2,4,6,7].

### 2.1 Eradicable immune responses: A formidable force

Eradicable immunological pathways are powerful defense mechanisms meticulously orchestrated to eliminate pathogens. These pathways are initiated by a specialized subset of T cells known as follicular helper T (Tfh) cells. Tfh cells are characterized by the expression of the CXCR5 chemokine receptor and the secretion of interleukin (IL)-21 [8,9]. Additionally, the transcription factors BCL6 and STAT5β play a crucial role in mediating these immune reactions [10,11]. The primary function of Tfh cells is to promote antibody production by B cells in the germinal centers of lymphoid tissues [8]. They also facilitate the class switch from IgM to IgG, a process mediated by IL-21. This class switch is critical for the activation of eradicable immune responses, as IgG antibodies are particularly adept at neutralizing and eliminating pathogens [12]. Within eradicable immune responses, four distinct branches have evolved to combat specific categories of pathogens: intracellular, parasitic, extracellular, and infectious.

### 2.2 TH1 immunological pathway: combating intracellular pathogens

The TH1 immunological pathway is the body's defensive line against intracellular microorganisms, such as bacteria, fungi, and protozoa. Key players in this pathway are type 2 myeloid dendritic cells, type 1 innate lymphoid cells (ILCs1), type 1 macrophages (M1), IFN-γ-producing CD4^+^ T lymphocytes, cytotoxic T lymphocytes, type 1 invariant natural killer T cells (iNKT1), and IgG3-secreting B lymphocytes. The TH1 immune response is activated by IL-12 and regulated by the transcription factors STAT4 and STAT1. The effector cytokine IFN-γ activates M1 macrophages via inducible nitric oxide synthase, leading to the generation of free radicals that initiate lipid membrane peroxidation, ultimately killing the ingested microorganisms. This pathway is associated with delayed type IV hypersensitivity reactions [2,4,13,14].

### 2.3 TH2 immunological pathway: combating parasitic pathogens

The TH2 immune route essentially defends host against parasitic threats and includes two subcategories: TH2a and TH2b. TH2a immunity targets endoparasites, such as helminths, while TH2b immunity targets ectoparasites, such as insects. In the TH2a immune response, Langerhans cells present antigens, and type 2 IL-25-producing natural innate lymphoid cells (nILCs2) are involved. Key interleukins in this process include IL-4 as well as IL-5, with STAT6 and STAT1 serving as crucial transcription factors. Major effector cells include inflammatory eosinophils, tryptase mast cells, IL-4/IL-5-producing CD4^+^ T lymphocytes, iNKT2 cells, and IgG4-producing B lymphocytes. In the TH2b immune response, Langerhans cells continue to present antigens, while type 2 IL-33-producing inflammatory innate lymphoid cells (iILCs2) are involved. Key cytokines here are IL-4 and IL-13, with STAT6 and STAT3 as the key transcription factors. Major effector cells are basophils, mast cell tryptase/chymase, IL-4/IL-13-producing CD4^+^ T lymphocytes, iNKT2 cells, and IgE-producing B lymphocytes. The TH2 route is associated with type I allergic autoimmune reactions, with TH2a and TH2b immunity related to IgG4-and IgE-dominant allergies, respectively [[1], [2], [3],^13^,^15^].

### 2.4 TH22 immunological pathway: combating extracellular threats

The TH22 immune route protects the body from extracellular microorganism such as bacterium, fungus, and protozoan. In this pathway, type 1 myeloid dendritic cells act as antigen-presenting cells, and type 3 NKp46+ innate lymphoid cells (NCR + ILC3) are the innate lymphoid cells involved. Effector immune cells in this pathway are PMNs, IL-22-producing CD4^+^ T lymphocytes, and IgG2-producing B lymphocytes. This pathway is activated by cytokines IL-1, IL-6, and tumor necrosis factor-alpha. The effector cytokine IL-22 is regulated by transcription factors STAT3 and STAT4. Neutrophil activation, including phagocytosis and neutrophil extracellular trap formation, effectively eliminates extracellular pathogens. Additionally, free radicals generated from PMN phagocytosis cause lipid peroxidation of cell membrane, leading to the death of these pathogens. This pathway is correlated to type III immune complex-mediated autoimmune reactions [1,2,[16], [17], [18], [19], [20]].

### 2.5 THαβ immunological pathway: combating infectious pathogens

The THαβ immune route protects the body from invasion of infectious pathogens, such as viruses and prions. Previously identified as type 1 regulatory cells (Tr1 cells), THαβ cells produce large amounts of IL-10. Although IL-10 is often considered immunosuppressive, it can stimulate natural killer and cytotoxic T cells and potently suppress pro-inflammatory cytokine production [21,22]. In THαβ immunity, plasmacytoid dendritic cells are the major antigen-presenting cells, and IL-10-secreting innate lymphoid cells (ILC10) are involved. Effector immune cells in this pathway include natural killer (NK) cells, IL-10-producing CD4^+^ T cells, CD8^+^ T cells (Tc2), and IgG1-producing B cells. This pathway is activated by type I interferons as well as IL-10, with IL-10 as the vital effector interleukin. The pathway is regulated by transcription factors STAT1, STAT2, and STAT3. NK cell-mediated antibody-dependent cellular cytotoxicity via IgG1 antibodies leads to apoptosis of virus- or prion-infected cells. During apoptosis, DNA fragmentation destroys viral DNA or RNA, and protein digestion by caspases degrades prions. This pathway is correlated to type II antibody-dependent cytotoxic autoimmune reactions [2,6,7,23].

### 2.6 Tolerable immune responses: A delicate balance

Although the body's immune system is designed to eradicate pathogens, complete eradication can be challenging and potentially harmful to the host. Under such conditions, the body initiates tolerable immunological pathways mediated by regulatory CD4^+^CD25^+^ T cells (Tregs) with less intense inflammatory cytokines [[1], [2], [3], [4]]. These FOXP3+ Tregs secrete TGF-β, which activates STAT5α and STAT5β to initiate tolerable immune route [24]. STAT5α and STAT5β have distinct, non-redundant roles in Treg induction [25]. Additionally, TGF-β induces B-cell antibody isotype class switching to IgA, a hallmark of tolerable immune responses. IgA1 and IgA2, subtypes of IgA, are primarily localized in the serum and mucosa, respectively. TGF-β can induce IgA1 or IgA2 production depending on the lymphoid follicle location [26,27]. Other immune cells associated with Tregs include DCregs, Bregs, and ILCregs. DCregs are antigen-presenting cells, while ILCregs are initial innate helper cells involved in Treg induction [28]. The tolerable immune routes are grouped to four key branches.

### 2.7 TH1-like immunological pathway: tolerating intracellular invaders

The TH1-like immune route represents the body's tolerable immunity path fighting intracellular microorganism, such as bacterium, fungus, and protozoan. Effector cells in this pathway include macrophages (M2), IFN-γ/TGF-β-secreting CD4^+^ T cells, CD8^+^ T cells, iNKT1 cells, and IgA1-producing B cells. TH1-like Foxp3+ CD4^+^ T cells secreting IFN-γ/TGF-β have also been identified [29,30]. Type 2 myeloid dendritic cells serve as antigen-presenting cells, while type 1 non-cytotoxic innate lymphoid cells are involved in this pathway. Regulatory cytokines in this pathway include IL-12 and TGF-β. TH1-like immunity is associated with chronic intracellular pathogenic infections, such as tuberculosis and leishmaniasis [31,32], and type IV delayed-type hypersensitivity reactions, including Crohn's disease [2,4].

### 2.8 TH9 immunological pathway: tolerating parasitic pathogens

The TH9 immune route represents a tolerable body immune route fighting parasites, including insects and helminths. Effector cells include regulatory eosinophils, basophils, mast cells (MMC9), IL-9-secreting CD4^+^ T cells, iNKT2 cells, and IgA2-producing B cells. Langerhans cells act as antigen-presenting cells, while thymic stromal lymphopoietin-induced type 2 cells are innate lymphoid cells. The driving cytokines for this pathway are IL-4 and TGF-β. This pathway is associated with type I allergic hypersensitivity reactions [3,[33], [34], [35], [36]].

### 2.9 TH17 immunological pathway: tolerating extracellular threats

The TH17 immune route represents the body's tolerable immunity fighting extracellular microorganism, such as bacterium, fungus, and protozoan. Key immune cells include PMNs (N2), IL-17-producing CD4^+^ T lymphocytes, iNKT17 cells, and IgA2-producing B lymphocytes. Antigen-presenting cells are type 1 myeloid dendritic cells, and type 3 non-cytotoxic innate lymphoid cells (NCR- ILCs3) are the innate lymphoid cells involved. The driving cytokines for this pathway are IL-6 and TGF-β. This pathway is associated with type III immune complex-mediated hypersensitivity reactions [2,16,[37], [38], [39], [40], [41]].

### 2.10 TH3 immunological pathway: tolerating infectious pathogens

The TH3 immune route represents the body's immunity fighting infectious molecules, such as viruses and prions. Plasmacytoid dendritic cells serve as antigen-presenting cells, and ILC10 cells are innate lymphoid cells. Effector immune cells in this pathway include natural killer cells (NK2), IL-10/TGF-β-secreting CD4^+^ T lymphocytes, cytotoxic T lymphocytes, and IgA1-producing B lymphocytes. Major effector interleukins are TGF-β and IL-10. IL-35-secreting CD4 T cells, also known as iTr35 cells, have been identified as a new type of T helper cells triggered by IL-10 and TGF-β, and are likely TH3 cells [[42], [43], [44], [45]]. Vital transcription factors of TH3 immune route include STAT1, STAT3, and STAT5α/β. This pathway is correlated to type II antibody-dependent cytotoxic autoimmune reactions [2,23].

### 2.11 Clonal anergy mechanism: distinguishing self from non-self

The core of the immune system's function is its ability to distinguish between foreign and self-antigens, a process known as clonal anergy. This mechanism ensures that immune cells do not mount an immune response against the body's own tissues, thereby preventing autoimmune disorders. Each T or B cell is specific to a single antigen, a phenomenon referred to as the clonal mechanism. When a clonal T or B cell encounters a self-antigen, it does not initiate an immune response; this state is known as clonal anergy. The mechanism underlying clonal anergy is mediated by gamma-delta T cells or immunoglobulin D (IgD) B cells. Gamma-delta T cells develop in the thymus earlier than alpha-beta T cells. Thus, a T-cell clone first differentiates into gamma-delta T cells when it recognizes a self-antigen. Consequently, the later development of T cells does not recognize self-antigens and only responds to foreign antigens. When gamma-delta T cells recognize self-antigens, they induce clonal anergy [4,[46], [47], [48], [49], [50]].

A similar mechanism is observed in B lymphocytes. Mature B cells coexpress IgD and IgM on their surfaces. When IgD on a B cell recognizes a self-antigen, it triggers clonal anergy without initiating an immune response. Conversely, when IgM on a B cell recognizes a foreign antigen, it prompts an immune route fighting the environmental pathogen. Subsequently, the IgM-bearing B lymphocytes will switch antibody isotype to IgG, IgE, or IgA. This finely tuned clonal anergy mechanism ensures that immune defenses are directed solely at foreign threats while maintaining tolerance to the body's tissues [[51], [52], [53], [54], [55]].

In summary, host immunological pathways represent a complex and intricate network meticulously regulated to protect the body from potential threats. IgG-dominated immune responses serve as formidable defense mechanisms against pathogens, while IgA-mediated responses manage chronic or severe infections with minimal host damage. These processes are further enhanced by the clonal anergy mechanism, ensuring that the immune system's potent defenses target only foreign threats. This comprehensive immunological framework exemplifies the remarkable adaptability and sophistication of the human immune system, reflecting its major evolutionary design.

## 3 Enigmatic role of tregs in organ fibrosis and failure

A delicate balance exists between eradicable and tolerable immune responses, each serving a distinct purpose. IgG-dominated eradicable immune responses represent the body's robust defenses, used to eliminate pathogens. In contrast, IgA-regulated tolerable immune responses maintain equilibrium by managing chronic or severe infections while minimizing damage to the host. At the core of these tolerable immune responses is a specialized subset of Tregs that orchestrate immunological processes with profound implications for organ health and function.

### 3.1 Tregs in systemic sclerosis

Systemic sclerosis, also known as scleroderma, is a debilitating autoimmune disorder characterized by widespread fibrosis of affected tissues. This Treg-dominant autoimmune condition often leads to multiple types of end-stage organ failure. This review discusses both scleroderma and end-stage organ failure to determine possible common pathophysiologies [56,57]. Scleroderma manifests in two major subgroups: diffuse and limited cutaneous. These subgroups are distinguished by the degree of trunk involvement. The limited cutaneous form often presents with a subset called CREST syndrome, which includes calcinosis cutis, Raynaud's phenomenon, esophageal dysmotility, sclerodactyly, and telangiectasia. Regardless of the subtype, scleroderma is defined by the replacement of normal tissue architecture with rigid acellular connective tissues. Fibrosis can affect multiple organs, including the skin, lungs, gastrointestinal tract, kidneys, and heart, leading to numerous functional impairments and potentially life-threatening complications.

Tregs play a pivotal role in the pathogenesis of systemic sclerosis, characterized by FOXP3 expression and the production of TGF-β, a potent mediator of tissue fibrosis [58]. TGF-β is often referred to as the “master regulator” of fibrosis because it stimulates fibroblast proliferation and promotes epithelial-mesenchymal transition, a process implicated in the development of scleroderma [[59], [60], [61], [62], [63]]. Evidence from mouse models and clinical trials underscores the significance of TGF-β in scleroderma [64]. Anti-TGF-β treatments have prevented skin and lung fibrosis in animal studies, while clinical trials have shown the potential of these therapies to alleviate symptoms of systemic sclerosis [64].

Polymorphisms in FOXP3, the major Treg transcription factor, are associated with the incidence of systemic sclerosis, highlighting the intricate link between these cells and disease pathogenesis [65]. The simultaneous presence of IgG and IgA in patients with scleroderma provides additional insights into the role of Tregs. IgG is associated with eradicable immune responses, whereas IgA is associated with tolerable Treg-induced immune reactions [66]. The presence of both antibody isotypes suggests that both eradicable and tolerable immune responses contribute to the pathogenesis of systemic sclerosis. 10.13039/100014337Furthermore, elevated levels of IL-2 and IL-35, which promote Treg activity, in patients with scleroderma further support their involvement in the disease process [43,67,68].

### 3.2 Tregs and heart failure

Heart failure, a debilitating condition marked by the heart's inability to pump blood effectively, can severely impact overall health and quality of life. Symptoms of heart failure include shortness of breath, excessive fatigue, and lower leg edema, while complications such as pulmonary edema, respiratory failure, and cardiac arrhythmias can be life-threatening. Chronic inflammation and subsequent cardiac fibrosis are major contributors to the development of heart failure. Tregs and the associated TGF-β signaling pathway play a crucial role in this fibrotic process. Tregs stimulate cardiac fibroblast proliferation and promote heart fibrosis [69]. Elevated TGF-β levels have been linked to cardiac remodeling and the progression of heart failure in animal models [70,71]. Furthermore, polymorphisms in the TGF-β gene have been associated with end-stage heart failure, highlighting its significance in the disease's pathogenesis [72,73].

The transcription factor SMAD7, which inhibits TGF-β signaling, has been shown to prevent post-infarction heart failure, suggesting potential therapeutic implications for targeting this pathway [74]. Additionally, microRNAs and other non-coding RNAs that interact with TGF-β influence cardiac fibrosis during heart failure, underscoring the intricate interplay between these regulatory mechanisms [75]. Tolerable immune responses initiated by Tregs, such as those involving TH17 and TH9, have also been implicated in the pathogenesis of heart failure. The central cytokines of these pathways, IL-17 and IL-9, respectively, are linked to cardiac fibrosis, remodeling, and progression of heart failure [76,77]. Moreover, coxsackievirus-induced myocarditis and subsequent heart failure are closely related to overt anti-viral immunity, such as the TH3 immune reaction [78]. Chagas disease, caused by the protozoan *Trypanosoma cruzi*, has a TH1-like pathogenesis leading to cardiomyopathy and subsequent heart failure [79]. Thus, all four tolerable immune reactions are linked to heart failure.

### 3.3 Tregs and liver cirrhosis

Liver cirrhosis, the terminal stage of liver failure, is marked by extensive fibrosis and the subsequent development of complications such as jaundice, esophageal varices, ascites, and splenomegaly. The primary causes of liver cirrhosis include alcoholic liver disease, non-alcoholic steatohepatitis, chronic viral hepatitis, and drug abuse. Chronic liver inflammation is a common pathway leading to cirrhosis, with TGF-β playing a critical role in the fibrotic process. Elevated levels of TGF-β have been observed in patients with liver cirrhosis and have also been implicated in the development of hepatocellular carcinoma, a malignancy often associated with cirrhosis [80].

Tregs and their associated tolerable chronic immune responses are key players in the pathophysiology of liver cirrhosis. Treg expansion has been noted in the acute decompensation of liver cirrhosis, as well as in cirrhosis induced by hepatitis B virus (HBV) infection and Schistosoma-related liver fibrosis [80,81]. HBV- and Schistosoma-induced liver cirrhosis are associated with anti-viral TH3 and anti-parasitic TH9 immune responses, respectively [80,82,83]. Additionally, Tregs have been implicated in primary biliary cirrhosis and other liver cirrhosis-related disorders [84]. Primary biliary cirrhosis is linked to TH17 and TH1-like inflammation [85]. Furthermore, IgA levels, which are associated with tolerable Treg-induced immune responses, are significantly elevated in patients with liver cirrhosis. The characteristic β-γ bridging pattern observed in serum electrophoresis, due to elevated serum IgA levels, is a hallmark of liver cirrhosis. IgA vasculitis and nephropathy have also been associated with the onset of liver cirrhosis, underscoring the link between IgA levels and this condition [86,87]. Moreover, TH17 immune responses initiated by Tregs are upregulated in liver cirrhosis. These findings highlight the intricate interplay between Tregs and organ fibrosis [81].

### 3.4 Tregs and uremia

Uremia, or chronic renal failure, represents the end stage of renal dysfunction. This condition is marked by the kidneys' impaired ability to eliminate metabolic waste products. Without intervention through dialysis or kidney transplantation, uremia can lead to life-threatening complications, including coma and death. Conditions leading to chronic renal failure include diabetes, hypertension, glomerulonephritis, interstitial nephritis, polycystic kidney disease, and recurrent urinary tract infections. Chronic inflammation is a common pathway contributing to the onset of uremia. TH1 and TH17 immune reactions are associated with diabetic kidney disease and may progress to uremia [88,89]. IL-9, a key cytokine in the TH9 immune reaction, contributes to the progression of chronic kidney disease (CKD) [90]. Similarly, hemolytic uremic syndrome can be induced by virus-triggered TH3 immune reactions, including infections by Epstein-Barr virus (EBV), adenovirus, and influenza virus [[91], [92], [93]]. IgA nephropathy, characterized by the presence of anti-IgA antibodies, often leads to chronic renal failure. IgA is associated with tolerable Treg-induced immune reactions, suggesting a potential link between Tregs and uremic pathogenesis [94,95].

TGF-β plays a crucial role in the progression of renal fibrosis, a hallmark of chronic renal failure [96]. It is implicated in the chronic progression of IgA glomerulonephritis and diabetes-related nephropathy [[97], [98], [99]]. Additionally, the expression of the TGF-β/Smad signaling pathway has been reported in children with IgA nephropathy, underscoring its significance in the disease's pathogenicity [95]. TGF-β-induced epithelial-mesenchymal transition, a process in which epithelial cells transition to a mesenchymal phenotype, plays a vital role in renal fibrosis [100]. Latent transforming growth factor beta binding protein 4, a regulator of TGF-β, has also been implicated in renal fibrosis pathogenesis [101]. Importantly, TGF-β/Smad inhibitors have shown potential in alleviating the progression of renal fibrosis, highlighting the therapeutic potential of targeting this pathway in managing uremia [[102], [103], [104]].

### 3.5 Tregs and pulmonary fibrosis

Pulmonary fibrosis, a progressive and debilitating lung disease, is characterized by the scarring and stiffening of lung tissue, leading to impaired respiratory function. Symptoms such as shortness of breath, dry cough, fatigue, and weight loss can significantly affect a patient's quality of life. Complications like pulmonary hypertension, respiratory failure, and pneumothorax can exacerbate the condition. The underlying causes of pulmonary fibrosis range from environmental pollutants and certain medications to autoimmune disorders, viral infections, and interstitial lung diseases. However, in many cases, the cause remains unknown, and the condition is termed idiopathic pulmonary fibrosis.

Chronic inflammation is a common pathway leading to lung fibrosis, with TGF-β being a key mediator in the pathogenesis of this condition [[105], [106], [107], [108]]. TGF-β has been detected at sites of extracellular matrix gene expression in human pulmonary fibrosis, suggesting its involvement in the fibrotic process [107]. Acute respiratory distress syndrome (ARDS), a potentially life-threatening condition following viral or bacterial infections [109,110], is a TH17-dominant inflammatory disorder associated with the development of pulmonary fibrosis. Furthermore, TGF-β has been implicated in the pathophysiology of this disease, highlighting its role in lung fibrosis [111,112]. Long-term uncontrolled asthma with a TH2 or TH9 immune response can lead to lung fibrosis [[113], [114], [115]], and lung remodeling with pulmonary fibrosis can be a sequela of tuberculosis infection, which induces a TH1-like immune reaction [116]. Viral infections, including EBV, CMV, HHV-7, and HHV-8, markedly increase the risk of idiopathic pulmonary fibrosis [117,118], indicating that virus-induced TH3 immune reactions may play a role in its pathogenesis.

Inhibition of TGF-β receptor signaling has prevented lung fibrosis in animal models, underscoring the potential therapeutic value of targeting this pathway. Additionally, microRNAs such as miR-133a, which inhibits TGF-β1-induced myofibroblast differentiation, have prevented pulmonary fibrosis in mouse models [119]. Tregs, through their production of TGF-β, play a vital role in the pathogenesis of pulmonary fibrosis. Inhibition of miR-182–5p, which attenuates pulmonary fibrosis via the TGF-β/Smad pathway, further supports the involvement of Tregs in this debilitating condition [120].

## 4 Interplay of end-stage organ damage: the heart, kidneys, lungs, and liver

The function of Treg cells in the pathophysiology of end-stage organ failure extends beyond individual organ systems, as evidenced by the intricate interplay between the heart, kidneys, lungs, and liver. Cardiorenal syndrome, a clinical condition that describes the coexistence of heart and renal failure, highlights the interconnection between these organ systems. Similarly, hepatorenal syndrome refers to the concurrent presence of liver and renal failure, while “hepatocardiorenal syndrome” encompasses interactions among the heart, liver, and kidneys [121].

**Fig. 1:**
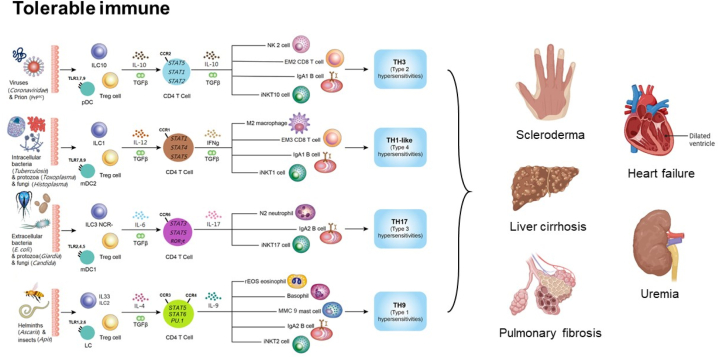
The framework of tolerable immune responses and their relationships to scleroderma, heart failure, liver cirrhosis, uremia, and pulmonary fibrosis. Tolerable immune responses are initiated by Treg cells and can be further categorized into TH3, TH1-like, TH17, and TH9 immunological pathways. The TH3 pathway mediates immune responses against viruses and prions. The TH1-like pathway targets intracellular bacteria, protozoa, and fungi. The TH17 pathway is responsible for immune responses against extracellular bacteria, protozoa, and fungi. Lastly, the TH9 pathway focuses on immune responses against parasites.

Treg populations are elevated in patients with cardiorenal syndrome compared to those with chronic kidney disease alone [122]. This observation suggests that Tregs may act as key mediators in the interactions between end-stage organ damage processes. Furthermore, the concept of cardiopulmonary–renal interaction underscores the complex relationships between these vital organs and the potential contribution of Tregs to the progression of multiorgan dysfunction [123]. The summary of Treg induced tolerable immune responses and their relations to systemic sclerosis and end organ failures is demonstrated in Fig. 1.

## 5 Therapeutic implications and conclusion

Several limitations in the current literature were noted: Additional studies on pathophysiological mechanisms other than TGF-β-induced fibrosis are needed, both *in vitro* and *in vivo*, to elucidate the roles of Tregs in the development of end-stage organ failure. Although four immunological pathways have been proposed for tolerable immune responses, further investigation is warranted to identify the central cytokines involved in TH3- and TH1-like immune reactions.

The enigmatic role of Tregs in organ fibrosis presents both challenges and opportunities for therapeutic intervention. By elucidating the common pathophysiological mechanisms underlying conditions such as systemic sclerosis, heart failure, liver cirrhosis, uremia, and pulmonary fibrosis, we can gain invaluable insights into potential therapeutic targets.

Tregs, through their production of TGF-β, emerge as key mediators of end-stage organ fibrosis. Consequently, strategies aimed at controlling chronic inflammation and the associated tolerable immune reactions may hold the key to preventing and managing these debilitating conditions.

Therapies targeting the TGF-β signaling pathway, such as TGF-β inhibitors or modulators of the associated signaling cascades, have shown promise in preclinical and clinical studies. Additionally, the regulation of microRNAs and non-coding RNAs that interact with the TGF-β pathway presents a novel therapeutic avenue.

Looking forward, preventing uncontrolled tolerable immune reactions through the overactivation of TGF-β-producing Tregs by avoiding organ inflammation is crucial. Harmful inflammatory responses can lead to organ failure, so preventing such inflammation can reduce the need for final treatment strategies like organ transplantation. Continuous overt inflammation can damage transplanted organs, resulting in loss of function. For example, ARDS can induce pulmonary fibrosis through excessive TH17 immunity, triggered by IL-17, IL-6, and TGF-β. Blocking these cytokines with antibodies or inhibitors may prevent the onset of pulmonary fibrosis, pulmonary hypertension, and respiratory failure.

Clarifying the intricate interplay between Tregs, TGF-β, and organ fibrosis can facilitate the development of effective treatments for related diseases and improve the quality of life for countless individuals worldwide.

## Data availability statement

This review was performed through a literature search without conducting experiments. Therefore, data availability is not applicable. No new data were generated in this review article.

## Ethical statement and consent

This review article explores the relationship between immune pathways and cell death mechanisms through a literature search. No experiments were conducted, and sources were drawn from PubMed and MEDLINE, eliminating the need for ethical statements and informed consent for the preparation and finalization of the article.

## Funding

This study was supported by grants from 10.13039/501100008108Taipei Tzu Chi Hospital, 10.13039/501100005925Buddhist Tzu Chi Medical Foundation (TCRD-TPE-110-02(2/3) and TCRD-TPE-111-01(3/3)).

## CRediT authorship contribution statement

**Kuo-Cheng Lu:** Writing – original draft, Resources, Methodology, Investigation, Formal analysis. **Kuo-Wang Tsai:** Writing – original draft, Investigation, Funding acquisition, Formal analysis. **Wan-Chung Hu:** Writing – review & editing, Validation, Supervision, Conceptualization.

## Declaration of competing interest

The authors declare that they have no known competing financial interests or personal relationships that could have appeared to influence the work reported in this paper.
